# Intelligent fluorescence image analysis of giant unilamellar vesicles using convolutional neural network

**DOI:** 10.1186/s12859-022-04577-2

**Published:** 2022-01-21

**Authors:** Il-Hyung Lee, Sam Passaro, Selin Ozturk, Juan Ureña, Weitian Wang

**Affiliations:** 1 Department of Chemistry and Biochemistry, Montclair State University, Montclair, NJ 07043 USA; 2 Department of Computer Science, Montclair State University, Montclair, NJ 07043 USA

**Keywords:** Image analysis, Fluorescence imaging, Cognitive neural network, Deep learning, Lipid membrane, Protein droplet, Phase separation, Vesicle analysis, Membrane reconstitution

## Abstract

**Background:**

Fluorescence image analysis in biochemical science often involves the complex tasks of identifying samples for analysis and calculating the desired information from the intensity traces. Analyzing giant unilamellar vesicles (GUVs) is one of these tasks. Researchers need to identify many vesicles to statistically analyze the degree of molecular interaction or state of molecular organization on the membranes. This analysis is complicated, requiring a careful manual examination by researchers, so automating the analysis can significantly aid in improving its efficiency and reliability.

**Results:**

We developed a convolutional neural network (CNN) assisted intelligent analysis routine based on the whole 3D z-stack images. The programs identify the vesicles with desired morphology and analyzes the data automatically. The programs can perform protein binding analysis on the membranes or state decision analysis of domain phase separation. We also show that the method can easily be applied to similar problems, such as intensity analysis of phase-separated protein droplets. CNN-based classification approach enables the identification of vesicles even from relatively complex samples. We demonstrate that the proposed artificial intelligence-assisted classification can further enhance the accuracy of the analysis close to the performance of manual examination in vesicle selection and vesicle state determination analysis.

**Conclusions:**

We developed a MATLAB based software capable of efficiently analyzing confocal fluorescence image data of giant unilamellar vesicles. The program can automatically identify GUVs with desired morphology and perform intensity-based calculation and state decision for each vesicle. We expect our method of CNN implementation can be expanded and applied to many similar problems in image data analysis.

**Supplementary Information:**

The online version contains supplementary material available at 10.1186/s12859-022-04577-2.

## Background

Giant unilamellar vesicle (GUV) is an artificial lipid membrane system characterized by its unilamellar lipid bilayer and micrometer-wide globular shape. Because GUVs have the advantage of deformability and a relatively large size that can be readily observed by optical microscopy, they have been used in a variety of biophysical and biochemical experiments [1]. Example studies include the study of lipid membrane phase separation [2–4], phase modulation by membrane anchored proteins [5, 6], reconstitution of membrane remodeling processes [7, 8], the viral assembly process [9], measurement of mechanical tension [10, 11], effect of osmotic pressure [12], membrane protein reconstitution [13], and monitoring protein binding to the membrane [14] to list a few.

Fluorescence imaging and fluorescence image analysis are extensively used in these types of experiments. Fluorescently tagged lipids are introduced so we can use fluorescence emission signal to observe the behavior of lipid membranes. The most common mode of analysis in GUV fluorescence imaging is manually studying each individual vesicle to examine enough vesicles for statistical analysis from a population of GUVs [5]. Although this may be suitable for experiments with a relatively small number of GUVs, for larger-scale sample numbers, manual analysis is very time consuming and often difficult to implement, particularly when researchers are not very experienced with the nature of the GUV experiments or when the lab does not have enough resources of labor to spend on the analysis. Automated analysis can provide an effective solution to these problems by standardizing the analysis procedure.

GUV image analysis is complicated enough that it requires well-designed algorithms to automate the analysis to the comparable level that can be performed by a trained researcher, and due to general interest in the field, there has been some effort to develop methodologies and software to automatically analyze the fluorescence GUV images [15–18]. Previous effort has been focused on segmentation of GUV from a single z-section image and calculating total fluorescence intensity on the membrane for the purpose of monitoring molecular binding interaction with the membranes. Hermann et al. introduced the circular Hough transformation (CHT) algorithm for the automated segmentation of GUV images for intensity computation in Matlab [15]. Sych et al., used a general particle detection segmentation and multi-color channel image stitching for intensity analysis of phase separated vesicles in the ImageJ [16].

Deep learning in image classification is a process in which a convolutional neural network (CNN) is trained with sample images, then the trained model can be used to recognize different images. It has been proven to be powerful in descriptor extraction and classification for visual recognition tasks [19]. Because of recent progress in deep learning, its capability and efficiency reach even beyond human recognition, and it has been applied to a wide range of related fields in leading-edge science and engineering [20–28]. Classifying the objects is a very common task needed in image analysis. The classification can be used to choose the desired objects after initial segmentation and it can be also used to classify the final images into categories of defined states.

In this report, we developed an improved software to automate the image analysis process using the whole 3D z-stack information. We developed methods to implement CNN to classify GUV images in the automated image analysis for the first time. We show three example cases of implementation based on the degree of CNN usage for the analysis. Program 1 is entirely computation based, program 2 uses CNN to select desired images to analyze, and program 3 uses CNN for the initial selection and for the final state decision. For each program, we share our experimental findings that were analyzed by each program. In the binding analysis, we show how his-tagged protein bound to Ni-DGS lipid can be detached by competitive binding of imidazole solution. GUV of certain lipid composition mimicking the composition of mammalian plasma membranes is known to show domain phase separation behavior [2, 5, 29]. In the phase state analysis, we present how the existence of a domain separated lipid phase can be detected by contour intensity analysis and CNN trained by virtually created fluorescence images. In addition to the GUV analysis, we show the case of analyzing cargo molecule concentration in the liquid phase-separated protein droplets. Proteins with multivalent binding interaction or intrinsically disordered domain interaction at a high concentration spontaneously form protein droplet domains that may serve as independent organelles in our cells [30–40]. In addition to the GUV analysis software development, we hope our work in this report can provide a comprehensive example to implement powerful deep learning approach in similar images analysis in science.

## Implementation

### Structure of algorithms

**Fig. 1:**
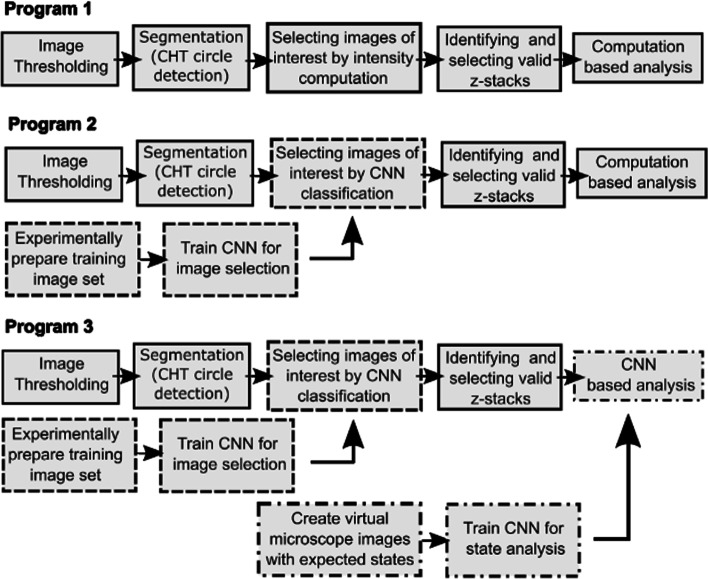
Structure of Algorithms. (1) Program 1: All processes done by pixel intensity based computation. (2) Program 2: Vesicle selection is replaced by CNN. (3) Program 3: Both vesicle selection and final state decision are done by CNN

We implemented our classification algorithms with three types based on the employment degree of CNN as shown in Fig. 1. Input data for the program are fluorescence image z-stacks, section images taken at different heights of the sample. We tested our program on confocal laser scanning microscopy images taken at 100 × optical magnification. The program first performs initial masking followed by segmentation to identify individual GUVs which appear as circular objects in the section images. Selection strategy is applied to choose only the vesicles of interest with desired morphology, and 3D GUV is identified based on the overall z-stack information. Valid z-stacks are chosen further analysis. Program 1 does everything computationally based on pixel intensity, program 2 uses experimentally trained CNN for vesicle selection, and program 3 uses experimentally and virtually trained CNN for vesicle selection and final classification of the outcome.

The CNN is mainly structured with the normalization layer (image input layer), the convolutional layer, the batch normalization layer, the rectified linear unit (ReLu) layer, the pooling layer, and the fully connected layer. During the training process, the sample images will be inputted to the normalization layer, which performs image normalization. The convolutional layer is applied for feature extraction. The batch normalization layer is used to normalize a mini-batch of data across all observations for each channel independently. The ReLu layer will introduce nonlinearity to the CNN model. The pooling layer will reduce the image spatial dimension. The fully connected layer is used for high-level reasoning in the neural network.

**Fig. 2:**
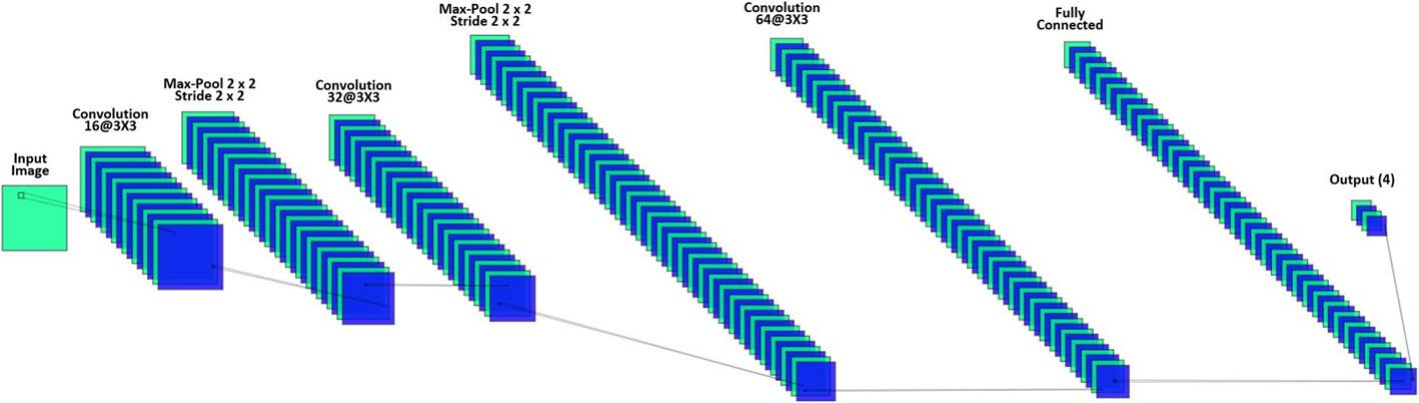
The CNN Architecture of in Program 2

In program 2*,* as shown in Fig. 2, the size of input images for the normalization layer is 50 × 50 × 1 images with 'zerocenter' normalization. Three convolutional layers are deployed in program 2. Convolutional layer 1 is a 2-D layer built with 16 filters of size 3 × 3 and 'same' padding, convolutional layer 2 is a 2-D layer built with 32 filters of size 3 × 3 and 'same' padding, and convolutional layer 3 is a 2-D layer built with 64 filters of size 3 × 3 and 'same' padding. In order to reduce the sensitivity to network initialization and speed up the training for CNN, we employ 3 batch normalization layers between each convolutional layer and ReLu layer. Two 2-D max pooling layers are used and each with the pool size as 2 × 2 and stride as 2 × 2. The output size of the fully connected layer is 4. A softmax function is used to normalize the output of the fully connected layer.

**Fig. 3:**
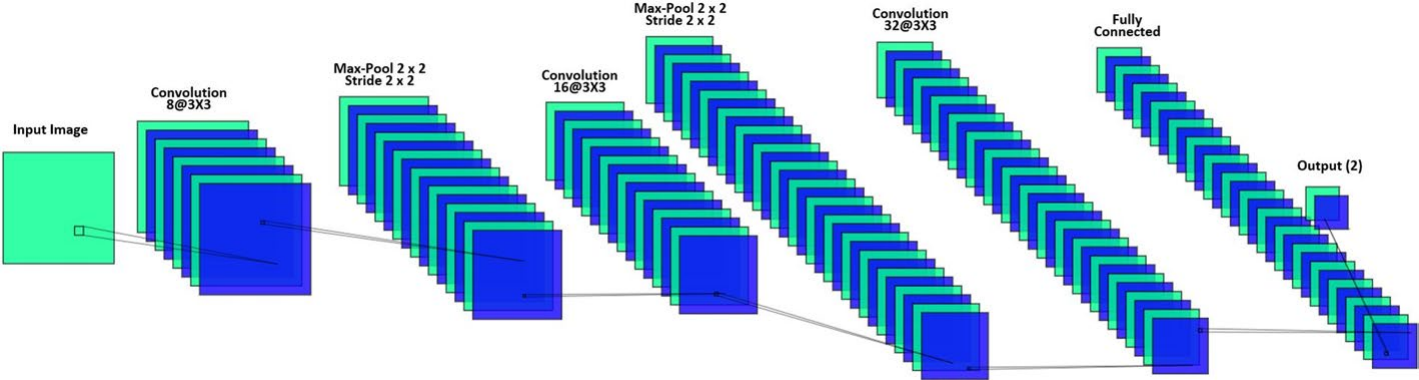
The CNN Architecture of in Program 3

In program 3, as presented in Fig. 3, the size of input images for the normalization layer is also 50 × 50 × 1 images with 'zerocenter' normalization. Three convolutional layers are deployed including: (1) convolutional layer 1 is a 2-D layer built with 8 filters of size 3 × 3 and 'same' padding, (2) convolutional layer 2 is a 2-D layer built with 16 filters of size 3 × 3 and 'same' padding, and (3) convolutional layer 3 is a 2-D layer built with 32 filters of size 3 × 3 and 'same' padding. We employ 3 batch normalization layers between each convolutional layer and ReLu layer. Two 2-D max pooling layers are used and each with the pool size as 2 × 2 and stride as 2 × 2. The output size of the fully connected layer is 2. A softmax function is used to normalize the output of the fully connected layer.

The stochastic gradient descent with momentum (SGDM) is used for both CNNs of program 2 and program 3. They have the same initial learning rate as 0.01. The maximum number of epochs for both CNNs is 4, where each epoch is a full training cycle on the entire training images. The data validation frequency of both CNNs is 30.

### Software platform

GNU Octave and MATLAB R2020b (MathWorks) with image processing and deep learning toolboxes were used to develop the software. We originally started the project with GNU Octave which is freely available, however, as we started to introduce CNN application, we needed accessible deep learning methods, and we changed our platform into MATLAB. Initial codes of program 1 are in GNU Octave and later codes of program 2 and 3 using CNN are in MATLAB.

### Image thresholding for high-sensitivity analysis of low signal-to-noise ratio images

We learned that creating a binary mask, which is an image where the original image is converted into 1 or 0 image based on the estimated threshold intensity, significantly enhanced the performance of segmentation, especially when the signal-to-noise ratio of the image is relatively low. To make the segmentation method generally applicable to images with any signal-to-noise ratio, initial binary masking was used. For typical GUV images, triangular thresholding estimation value worked the best although it is not the only possible thresholding for the purpose [41].

### CHT-based segmentation

Hermann et al. in 2014 introduced the circular Hough transformation (CHT) algorithm for the automated detection of GUVs in fluorescence images [15]. CHT is an algorithm originally developed to detect circular objects from complex images in computer science [42]. Briefly, CHT first converts the original image into an edge image to clearly define the edge of objects. Basing on the edge image, it starts the voting process to score the likelihood of having a circle with radius *r* at any given pixel position. The pixels at the center of circular objects appear as high-score pixels in the transformed image space; thus, the position of circles with radius *r* can be decided based on likelihood. If radius *r* is to be flexibly decided, radius *r* also becomes a parameter of the likelihood voting process, which makes it computationally more expensive. Fluorescence images of GUVs usually show very clear boundaries of circular contours due to membrane tension, so for most of the experiments that do not involve severe deformation of the vesicular membranes, CHT is an excellent method for segmentation. This segmentation could be replaced by a general object segmentation, but in most of our experiments, circular detection had an advantage in defining following computations.

### Selection filter and GUV stack analysis

After segmentation of GUVs, the program applies selection filters in order to choose GUVs with the desired morphology. In fluorescence imaging, the images of 3D objects are typically collected as z-stacks or a series of image sections at different heights. In some simple analyses, examining single section images may be sufficient, but whole stack analysis ensures that we are studying the entire 3D information of GUVs, and some filtering strategy and analysis are only possible when we have access to the whole 3D information.

**Fig. 4:**
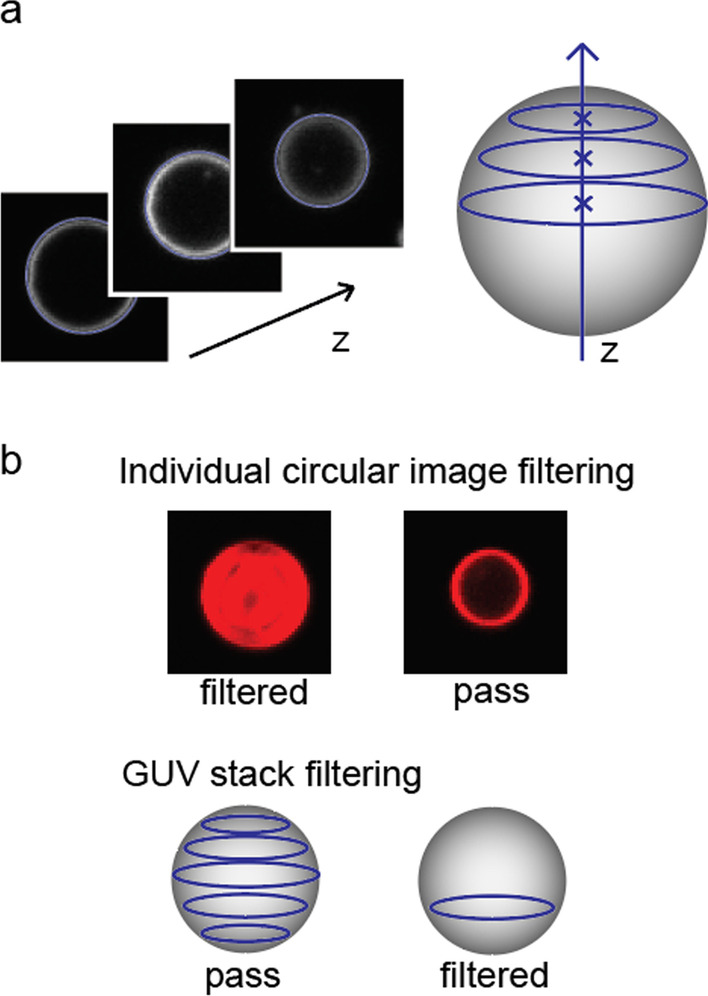
Schematic of GUV stack analysis. **a** Individual section images at different heights, or z positions are combined to construct a single whole GUV stack. The (x, y) positions of the centers are used to determine whether the z-section images are from the same vesicle. **b** Filtering strategies can remove vesicles that do not strictly meet the quality to proceed with the final analysis. Individual circular image filtering makes decisions based on the quality of each circular section image. For example, a vesicle with too much multi-lamellar intensity can be removed. GUV stack filtering makes decisions based on the quality of a GUV stack as a unit, such as an insufficient number of images, to reconstruct a whole GUV

Z-stacks are analyzed from the first to the last stack images one by one, as shown in Fig. 4a. In each image, the segmentation detects GUV fluorescence circles. The sensitivity parameter of CHT detection can be adjusted to change the strictness of circle definition, and the radius range can also be set to only detect GUVs with a reasonable size. GUV size from 20 to 120 pixels radius worked very well in any combinations of parameters. This covers most of the typical GUV sizes spanning tens of µm diameter in our imaging condition used. Once all GUV fluorescence circles are detected from each section of the image, 3D GUVs are identified by grouping fluorescence circles by their center positions. All fluorescence circles with center (x, y) positions within the set range are considered as one GUV and are grouped as an individual GUV entity. Each grouped GUV is recorded as a matrix containing the position, radius, and intensity of each image section that can be used for further analysis.

GUV selection strategies are applied at two separate stages, as shown in Fig. 4b. The first filtering is applied right after segmenting GUV fluorescence circles from each image. Only the GUV images with desired morphology are selected. In computation based selection strategy for program 1, the contrast between the circular edge and the internal space of the vesicle is used to filter out ill-defined circles. The intensity per pixel is calculated for pixels near edges and pixels inside the circle to compare, and any circles with insufficient relative intensity near edges are filtered out. This strategy can rule out vesicles with too low signal or too much fluorescence inside the vesicle such as multi-lamellar vesicles when we want to only analyze strictly unilamellar vesicles. GUV circles that are not clearly analyzable using the intensity contour because of ambiguous positions can be removed in this stage, as well. The second filtering strategy is done after grouping individual GUVs. In this stage, any GUVs with insufficient information can be excluded. Specifically, GUV groups with only one or two image sections are filtered out because they do not have enough images to be considered as an intact 3D GUV.

The result of the initial segmentation and filtering is displayed and recorded visually, so users can adjust the selection parameters to use until the outcome shows the most reliable detection result for the purpose of analysis.

### Intensity binding analysis

The purpose of intensity binding analysis is to quantify the change in fluorescence intensity on the membrane in a fluorescence channel for any membrane binding species. Reference channel A, in which the fluorescence signal comes from the lipid membrane, is used to detect and group GUVs to analyze; analysis channel B, in which the fluorescence signal comes from interacting species, such as membrane binding proteins, is analyzed for changes in the fluorescence intensity signal on the membrane.

**Fig. 5:**
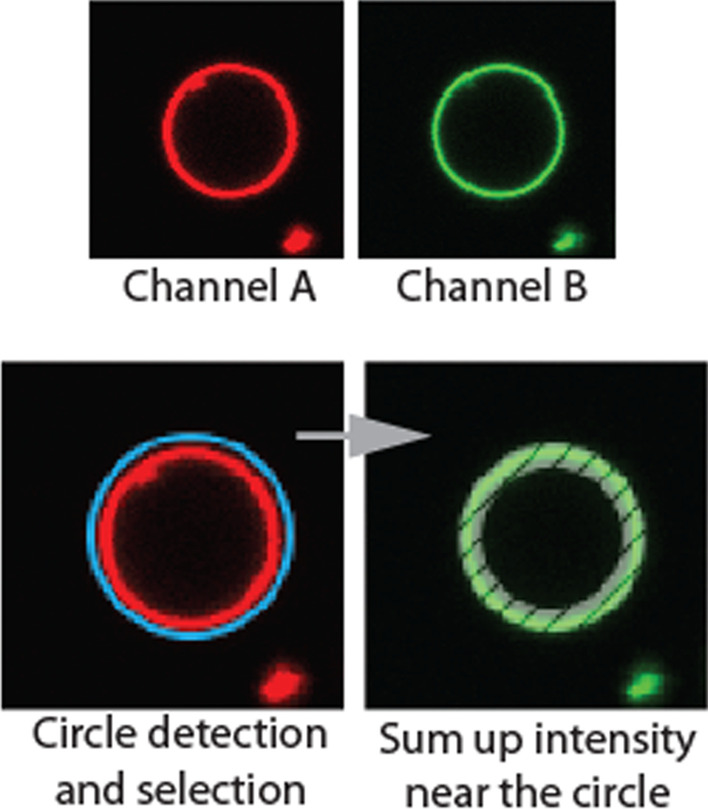
Circle detection and fluorescence intensity sampling. The channel A fluorescence signal comes from the fluorescently tagged lipid molecules. The channel A signal is used to detect circles. Using the information on the position and radius of the detected fluorescence circles from channel A, the matching signal from channel B is analyzed. The intensity signal of channel B comes from fluorescently tagged proteins that are supposed to bind to the lipid membrane. The net intensity of pixels that are close to the lipid membrane is summed up

For all fluorescence images that passed selection strategies, the matching fluorescence intensity of channel B is analyzed using the position and radius information obtained from channel A. The fluorescence intensity of all pixels adjacent to the circle defined by the center position and radius is summed up, as shown in Fig. 5. The total intensity values and intensity per pixel values are recorded for all images. Counting pixels that are *n* pixels, by user definition, inward successfully sums up intensity values. As each GUV is grouped, the average intensity per pixel from each GUV can be calculated.

To account for the background signal, the net intensity is defined as follows in Eq. 1:

1 $$I_{net} = I_{raw} - I_{background.}$$

This means that the general background signal per pixel was estimated and subtracted from the raw intensity signal. The background signal can be estimated by measuring the average signal coming from the dark part of the images. To make this process more systematic, we introduced automated methods to the program. In the automated estimation, a background signal is estimated based on the analysis of the intensity histogram of each image similar to the thresholding strategy for the initial segmentation.

### Phase domain separation state determination analysis using intensity contour computation

**Fig. 6:**
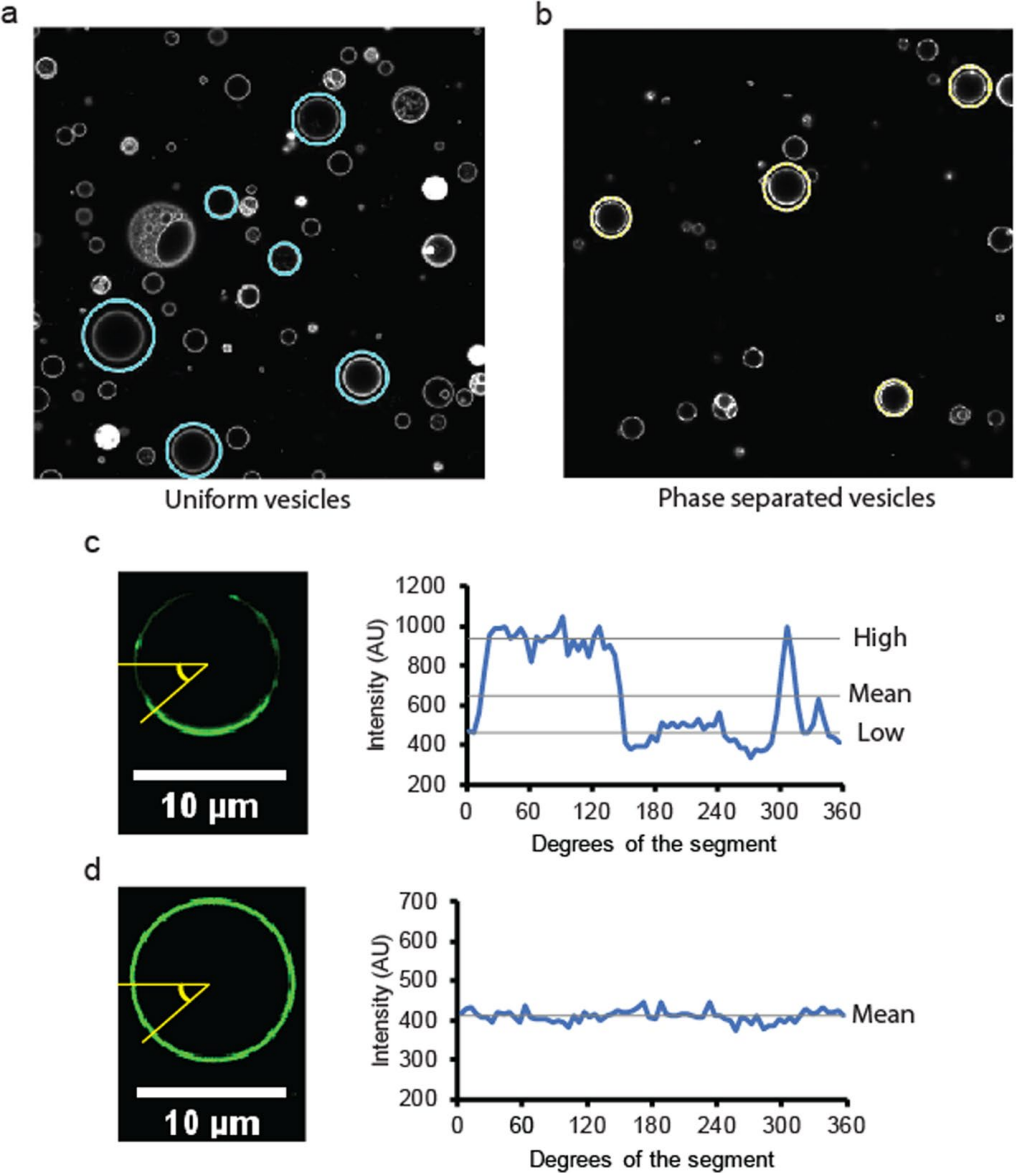
Contour intensity analysis for the determination of phase separation state. **a** A typical example of GUV detection from a uniform vesicle sample. Blue circles indicate the detected GUVs classified as uniform or no-phase domains. The composition of the vesicle was DOPC:Ni-DGS = 90:10 with 0.2 mol% of TopFluor-Cholesterol replacing the DOPC. **b** A typical example of GUV detection from a phase-separated vesicle sample. Yellow circles indicate GUVs classified as phase separated or the coexistence of phase domains. The composition of the vesicle was DOPC:Ni-DGS:DPPC:Cholesterol = 15:10:50:25 with 0.2 mol% of TopFluor-Cholesterol replacing the cholesterol. The images shown in a and b are 127.3 µm in width and height. **c** An example intensity trace along the perimeter of a phase-separated vesicle. The intensity trace shows clear discontinuity at the boundaries of two-phase domains, which can be used to determine the existence of phase domains. The average intensity in each domain can also be estimated from the intensity analysis, as shown. **d** An example intensity trace along the perimeter of a uniform vesicle. The intensity trace shows no detectable discontinuity, which suggests the nonexistence of phase domains within the vesicle

The purpose of phase separation determination analysis is to systematically identify the binary phase state of GUVs, homogeneous or domain separated, using a well-defined definition for the statistical analysis of a population of GUVs. Homogeneous vesicle show uniform intensity distribution while phase domain separated vesicles show two coexisting fluorescence intensity domains (Fig. 6). In addition to the phase determination, the analysis also calculates and reports the contour intensity of each z-section image of vesicles and the fluorescence intensity of different domains that may be used to calculate the partition coefficient of the reporter molecule.

Figure 6a, b show circular image segmentation for a typical uniform GUV sample with most of GUVs in a uniform phase state and a phase-separated GUV sample with most of GUVs in a phase-separated state. Program 1 and 2 use intensity contour computation for phase separation state decision. Intensity contour analysis uses the intensity around the circular perimeter of each section image to make a decision about phase separation. If the GUV is phase separated, the intensity around the perimeter will show discrete discontinuity at the phase domain boundary, as shown in Fig. 6c. If the GUV is uniform without phase separation, the intensity plot will not have any noticeable discontinuity around the perimeter, as shown in Fig. 6d. As this decision is made for each section of a GUV, the decision parameter can be set to make a final state decision for each GUV. For example, a GUV may be determined as a phase-separated vesicle when at least 40% of the z-section images show clear phase separation.

For contour intensity analysis, the intensity around the perimeter was divided into N segments. The average net intensity per pixel was calculated for each segment by using the pixels within (r-∆r, r) and (θ, θ + 360°/N), where r and θ indicate the radius and the angle in a polar coordinate of a circle image, respectively. For each contour intensity segment, histogram analysis is performed by aligning segments by average intensity from the highest intensity to the lowest one. The segment intensity of the 20% from the highest is defined as high intensity, and the segment intensity of the 20% from the lowest or 80% from the highest is defined as low intensity. These are estimated intensities of the fluorescence intensity of two different phase domains. The mid-intensity between the high intensity and the low one is calculated, and discontinuity analysis is performed along the direction of the increasing degree θ to determine whether there is any directional decrease or increase in intensity spanning ± p % from the mid-intensity (Fig. 6c, d). The p value may be adjusted based on the partition coefficient of the fluorescent report used. For a high partition coefficient reporter, which strongly prefers one domain over the other, a higher p may be used. For a low partition coefficient reporter with a relatively even distribution but with a clear preference for one domain over the other, a smaller p value should be used. High and low intensity estimation from the 20% percentile may be adjusted if necessary, but choosing a too small percentile is not recommended, as it may select a false high or low intensity coming from an outlier intensity. Choosing an overly large percentile may fail to correctly select high and low intensity in the histogram distribution when the relative proportion of one domain is small. Through an analysis of the number of discontinuities in the intensity contour, the number of domains may be estimated if needed, when finely modulated phase behavior with many domains per vesicle is expected, as opposed to a binary segregation with only two domains.

### Protein droplet intensity analysis

This analysis was performed as a possible application of program 1 to related samples. Protein droplet phase separation is another phase separation behavior that is commonly studied with an in vitro reconstitution experiment. Because of the comparable size and globular shape of the protein droplets to the GUVs, a few to tens of µm diameter, the same analysis method can be applied to automatically calculate the fluorescence intensity of each droplet. Segmentation can detect circular images from z-section images, and it can be reconstituted to find individual droplets. The fluorescence intensity of each droplet is analyzed by calculating the net average intensity per pixel of all the pixels within the circles that can be used to quantify the amount of protein in the droplet [31]. Background intensity is subtracted to correctly calculate the net intensity of the droplets, as specified in Eq. 1.

### CNN-based image filter for vesicle selection

The purpose of a CNN-based image filter is to enhance the capability of the GUV image selection strategy so that the program can recognize even more complicated cases of images that a simple calculation algorithm might miss. The CNN, a powerful and reliable network of deep learning, was used in program 2 and 3 to achieve this objective. Using the training images that were obtained and annotated experimentally, the CNN was trained by minimizing the mean squared error between the inputs and outputs. A total of 5853 training images of individual vesicles were used. The images were converted into 50 × 50 pixels as inputs for the CNN. Each image represented a possible z-section image of a GUV. The images were classified into four classes. In the training process, these four classes were defined as C1–typical unilamellar vesicles, C2–multi-lamellar vesicles, C3–vesicles overlapped with other vesicles, and C4–hazy or unclear images. A total of 1,918 training images of C1, 877 of C2, 1,129 of C3, and 1,929 of C4 were used. The neural network was trained and validated using the training set. Then, the trained CNN model takes unlabeled images as the input and generates the corresponding classes. In this version of the phase determination program, each detected vesicle image was converted into 50 × 50 images for classification by the trained neural network. Only images of C1–valid vesicles passed the enhanced filtering strategy. When training images were collected using the identical fluorophore and with similar optical condition, training the CNN with raw images of the specified numbers was enough to obtain reliable performance to select only the desired unilamellar vesicles. For the example networks trained by this method, CNN of program 2 reached 86.5% accuracy which was typical accuracy reached with experimental images using the conditions specified.

### CNN-based phase domain separation state determination analysis

In program 3, the final decision of vesicle phase decision was also done by trained CNN. The purpose of introducing CNN in the decision is to implement intensity computation free analysis that can potentially make decision for even more complicated variations of vesicle phase states that are tricky to distinguish based on computation. The CNN structure for state decision is very similar to the CNN for vesicle selection with 50 × 50 images as input. However, even though this task is relatively straightforward to human eyes, it turned out to be relatively complex task to train the CNN when we tried various CNN training. Experimental input of 1000 was not sufficient to get a reliable performance. To create enough number of training images, we used virtual images. In this method, virtual ground truth images of various phase states are created with randomized parameters and are automatically annotated to create images. GUVs are defined as a perfectly globular vesicle and domain separation is defined by overlapping a secondary globular shape within which is defined as a different domain. The ground truth images are then converted into virtual confocal fluorescence images by virtual noise addition and gaussian convolution. The general method of virtual confocal image creation was adopted and modified from Dmitrieff et al. [43]. For this specific task, we learned that eliminating the background intensity significantly improves the performance of the trained neural network, so image thresholding is performed for training images and also target analysis images. CNN training included training set augmentation of image scaling and position shifting. A total of 46,887 training images of C1 and 34,200 of C2 were used to train the network. In CNN based decision, phase state decision for each z-section image is replaced by the CNN base decision and the overall decision for a 3D GUV is determined the same way based on the set threshold number. For the example networks trained by this method, CNN of program 3 reached 99.9% which was typical accuracy reached with virtual images using the conditions specified.

### User parameters for the programs

Supporting document (Additional file 1) describes range of values assessed and recommended instruction to change all the user parameters used in the programs.

### Experimental sample preparation

GUV was prepared with the electroformation [44, 45] and gentle hydration [5]. Briefly, for electroformation, a lipid mixture of a desired composition was deposited on an indium tin oxide (ITO)-coated glass (Delta Technologies) at 55 °C. The lipid was dried by nitrogen gas and further dried in a vacuum chamber. The glass, along with another ITO glass, formed a sealed chamber separated by a silicon spacer (Grace Bio-Labs), and sucrose solution was introduced. Sinusoidal voltage of 2 V/mm space at 5 Hz was applied for about 2 h at 55 °C to induce GUVs. For gentle hydration, a lipid mixture is dried and vacuum incubated in a clean round bottom flask. Sucrose solution is added to incubate at 37 °C overnight. The GUVs were collected and centrifuged to remove large aggregates. Lipids used in this study were from Avanti Polar Lipids. The proteins utilized in this research were purified by *E. coli* overexpression, followed by Ni affinity purification and gel filtration. The plasmids were given by Michael Rosen (Addgene plasmid # 127093, #126946, and #127093) [31]. The imaging samples were prepared similar to the method described in [5]. Briefly, an AttoFluor cell chamber (Invitrogen) and a cover glass cleaned by bath sonication in isopropyl alcohol:water = 1:1 were assembled. The glass surface was blocked by incubating with 5 mg/mL BSA solution for 30 min. After incubation, the chamber was washed five times with the buffer for the addition of the GUV solution for imaging. To analyze protein interaction, the desired concentrations of proteins were added by pipette injection and mixed gently. To ensure a quick and homogeneous interaction, the protein solution was added at a volume comparable to that of the solution in the chamber (20%–50% by volume). Protein droplet samples were first mixed in a test tube to incubate for at least 30 min, and they were introduced to the chamber for imaging. To decrease the volume of the chamber, a silicon O-ring was introduced.

### Imaging condition

All the images shown in this report were collected using confocal fluorescence laser scanning microscopy unless specified otherwise. Briefly, a Nikon Ti-E-based C2 confocal microscope was used. Excitation laser lights of 488 and 561 nm were used with matching emission filters to collect signals from the fluorescent molecules. A Nikon Plan Apo 100 × NA 1.45 oil immersion objective was used without further magnifying the lens in the optical path. The typical mode of scanning was to collect data as 1,024 × 1,024 pixels spanning a 127.3 µm × 127.3 μm area, whereas motorized z-axis movement allowed the automated acquisition of z-stack images. All the example images shown and tested were collected by taking z-stack every 1 µm apart which is typically a great sampling in z-direction considering the resolution of confocal laser scanning and size of the GUVs. It means 1 µm/each z-section sampling resolution although optical resolution in z-direction in each image will be determined by the point spread function of the system. Images sampled at closer z distance can be analyzed the same way without any problems. Images sampled at greater z distance can be analyzed too, but caution should be taken to make sure there are enough number of z-stacks to define GUV entities.

## Results

### Program 1: Detecting the unbinding of his-tagged fluorescent proteins from the membrane by imidazole inhibition

**Fig. 7:**
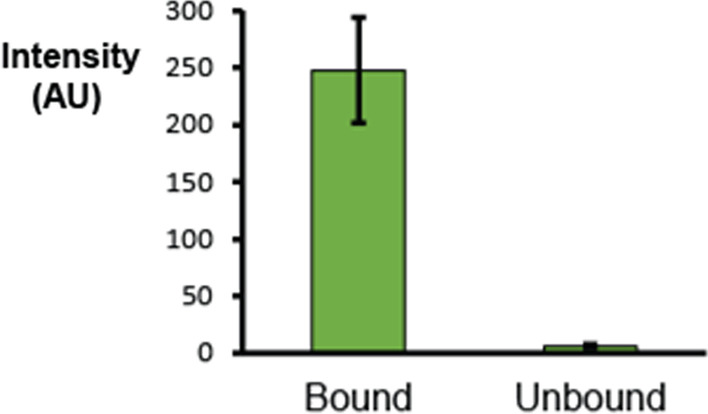
Results of the example data analysis by the automated intensity trace calculation. The fluorescence signals from the membrane-bound GFP proteins were quantified before and after the introduction of an inhibitor to detach the proteins from the membrane. The decrease in GFP intensity on the membrane is clear. Each average was calculated from 90 < n GUV samples analyzed from seven image stacks. Error bars indicate standard deviations between image stacks. The difference is statistically significance with *P* < 0.0001

We tested multi-channel intensity-based binding analysis by examining data from a binding/unbinding experiment of fluorescent proteins on the GUV membrane. In this experiment, his-tagged green fluorescence protein (GFP) was bound to the GUV with functionalized Ni-DGS lipids by incubating the sample with the protein. The protein was then detached by introducing a high concentration (300 mM) of imidazole, without altering the osmolality of the buffer, to detach the proteins from the membrane via inhibition of Ni–his tag interaction. We analyzed GFP fluorescence intensity on the membrane before and after introducing the inhibitor. Lipid and protein fluorescence signals were optically separated using different excitation and emission wavelengths. The program found circular fluorescence using the Texas Red-1,2-Dihexadecanoyl-sn-Glycero-3-Phosphoethanolamine (TR-DHPE) fluorescent lipid signal, and qualifying GUVs after selection strategies were used to quantify the fluorescence signal from the GFP protein on the membrane in the protein fluorescence channel. Multiple GUVs under the same conditions were quantified, and the average value was calculated. As shown in Fig. 7, the intensity-based binding analysis successfully quantified the difference before and after introducing the imidazole inhibitor to the sample. The amount of his-tagged GFP protein on the membrane after introducing the inhibitor was less than 5% of the intensity before introducing the inhibitor, suggesting that the majority of proteins that were originally bound to the membranes were removed from the membrane as a result of inhibition.

### Program 1: Protein droplet intensity analysis at different cargo concentrations

**Fig. 8:**
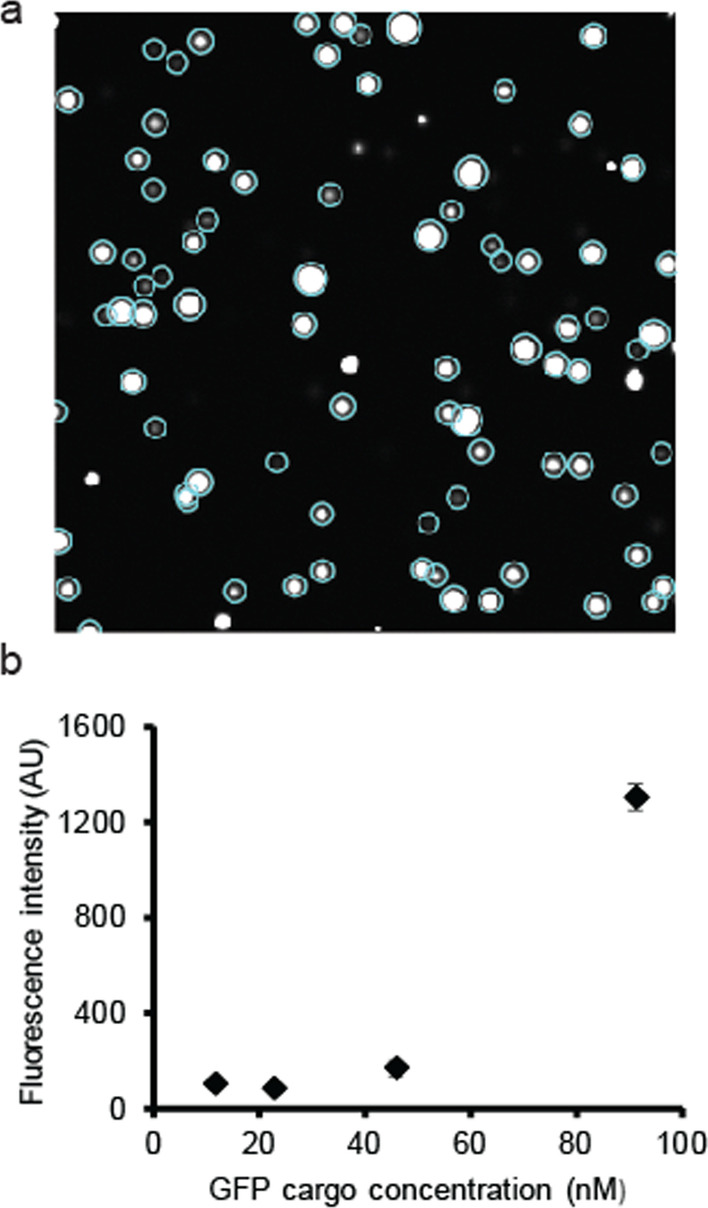
Automated protein liquid–liquid phase-separated droplet analysis. **a** CHT circle detection, followed by individual droplet grouping, efficiently detects well-behaved protein droplets from the sample. Blue circles indicate qualified protein droplets detected by the program. The image is 127.3 μm in width and height. **b** The average fluorescence intensities per pixel within droplets were quantified for the same protein droplets at different fluorescent cargo protein concentrations. The fluorescence signal increases as the cargo concentration increases, although there was only a negligible deference between two data points below 25 nM. Each average was calculated from n > 200 droplets from seven image stacks. Error bars indicate standard deviations between image stacks

We tested the protein droplet intensity analysis program by analyzing protein droplet samples prepared at different concentrations of fluorescent cargo proteins. As shown in Fig. 8a, the same approach of the automated analysis of CHT-based segmentation, followed by the whole stack analysis, selected individual protein phase droplets very effectively. The intensity was calculated for the entire area inside the circles instead of just the periphery for this calculation. The example analysis was performed on protein droplets formed between two multivalent binding partners, SUMOx10 repeats and SIMx10 repeats, at about 10 µM concentrations each. GFP-SUMO3, a fluorescent protein with three repeated SUMO domains, was introduced as a cargo protein that reported the protein droplet clearly. The cargo protein concentration was varied at a relatively low concentration range from 10 to 100 nM. At all concentrations, droplet detection was efficient, and fluorescence intensity showed a monotonic increase from 20 to 100 nM concentration regions, although it did not show a noticeable difference in intensity from 10 to 20 nM. (Fig. 8b).

### Program 2: CNN-based GUV selection strategy for enhanced vesicle identification

**Fig. 9:**
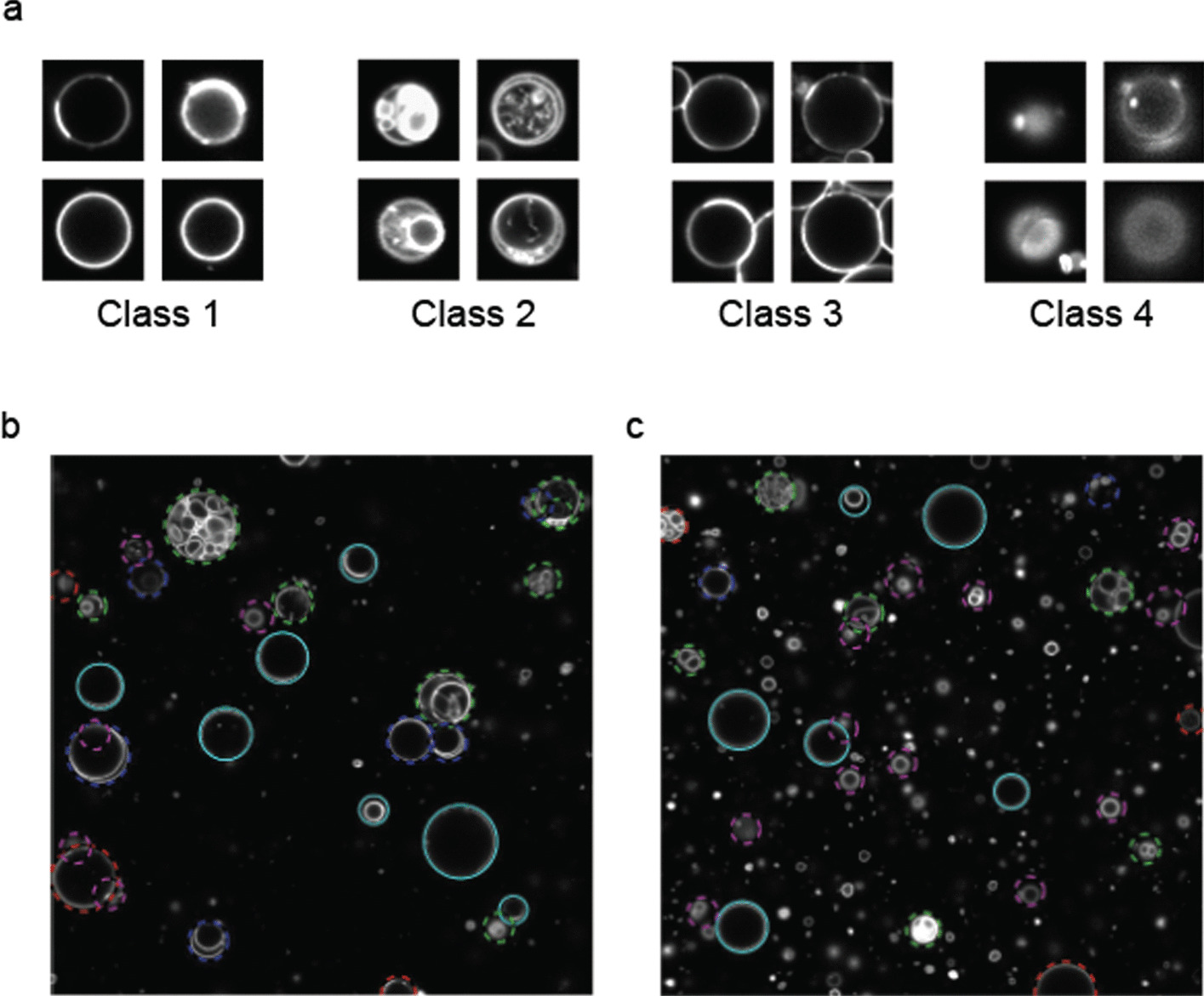
CNN-based vesicle selection filter. **a** Typical vesicle images used to train the neural network. Class 1 represents vesicles that are suitable for analysis. Class 2 represents multi-lamellar vesicles. Class 3 represents vesicles that are too closely overlapped with other vesicles, and class 4 represents vesicle images that are too hazy to analyze. **b**, **c** Example results of deep learning-based vesicle selection. Class 1—cyan, Class 2—green, Class 3—blue, Class 4—magenta, vesicles at the edges—red. Invalid vesicles were identified successfully for exclusion. The images are 127.3 μm in width and height

The deep learning-based approach uses CNN trained by the sample images. Program 2 image selection was done by the trained CNN. The sample images consist of pre-classified classes of images as the input. The CNN is trained until it can successfully classify images that were not part of the training sample images. This approach uses artificial intuition by pattern recognition of the objects, so it is potentially more capable when the objects to classify are difficult to define with a simple calculation-based strategy. Figure 9a shows the typical images in each class of images we used to train the neural network. Class 1 represents the desired vesicles to analyze; other classes represent vesicles that we do not want to analyze due to their ambiguity. Class 2 represents mutilamellar vesicles or vesicles that contain unexpected lipid membrane structures inside; class 3 represents vesicles that are too crowded, which makes the analysis of an individual vesicle ambiguous; and class 4 represents vesicles with a too low image quality to analyze mostly because the contour of the lipid membrane is not fully in focus.

When the trained network was used to detect vesicles images, the filtering strategy very strictly excluded vesicles that had any features represented by classes 2–4, as shown in Fig. 9b, c. Some vesicle images had multiple features of classes 2–4, such as a multi-lamellar vesicle with a hazy intensity trace. Therefore, some vesicles could not be classified uniquely, but the vesicles to be filtered almost always fell under one of classes 2–4, allowing only desired vesicle images of class 1 to survive the selection strategy. Combined with the whole z-stack approach, the likelihood of unwanted vesicles surviving the automated selection strategy was very small, and GUVs with desired morphologies were successfully selected. This approach was especially beneficial to exclude some cases that were ill defined by simple calculation. Multi-lamellar vesicles that have multilayers only near the outermost vesicle membranes and crowded vesicles in which a part of the membrane is touching other lipid membranes are examples.

### Program 2: Statistical analysis of phase state determination by intensity computation

We tested the contour intensity-based phase state determination program by analyzing samples with relatively clear differences in phase domain separation behavior. GUVs with uniform phase were prepared as a 1,2-dioleoyl-sn-glycero-3-phosphocholine (DOPC): 1,2-dioleoyl-sn-glycero-3-[(N-(5-amino-1-carboxypentyl)iminodiacetic acid)succinyl] (Ni-DGS) = 90:10 mol composition; 0.2 mol% of DOPC was replaced by TopFluor-labeled cholesterol (TF-Chol), a fluorescence reporter. GUVs with a highly domain-separated state were prepared as a DOPC:Ni-DGS: 1,2-dipalmitoyl-sn-glycero-3-phosphocholine (DPPC): Cholesterol = 15:10:50:25 mol composition; 0.2 mol% of chol was replaced by TF-Chol, a common fluorescence reporter of the same mol composition in both sets of GUVs. With an appropriate adjustment of parameters to use in the automated analysis, the program successfully detected and statistically distinguished two phase separation states, as shown in Table 1. To the best our knowledge, there hasn’t been any published software specifically designed for this purpose, so we compared the program performance with manual human decision. As shown in the result, when n > 100 vesicles from more than 20 images were analyzed, intensity calculation based state decision showed the comparable level of performance to human decision for the task. Mean accuracy was an average of % agreement between manual analysis and computer analysis for each vesicle decision. Individual decisions were visualized by saving color-coded images during the analysis, and we could review the saved images to validate the reliability of the state decision for each individual vesicle. Participating researchers were students with experience in fluorescence imaging, and the researcher who trained the network did not participate in this survey to avoid overfitting.

**Table 1:** Performance of state determination by intensity computation

Type	Total number of GUVs analyzed (n)	Number of GUVs domain separated	Number of GUVs uniform	Mean accuracy of state decision (%)
Researcher 1	113	49	64	(Reference)
Researcher 2	113	47	66	(Reference)
Researcher 3	113	49	64	(Reference)
Researcher 4	113	49	64	(Reference)
Researcher 5	113	50	63	(Reference)
Computation Analysis	113	49	64	99.47

### Program 3: CNN-based analysis of phase state determination

**Fig. 10:**
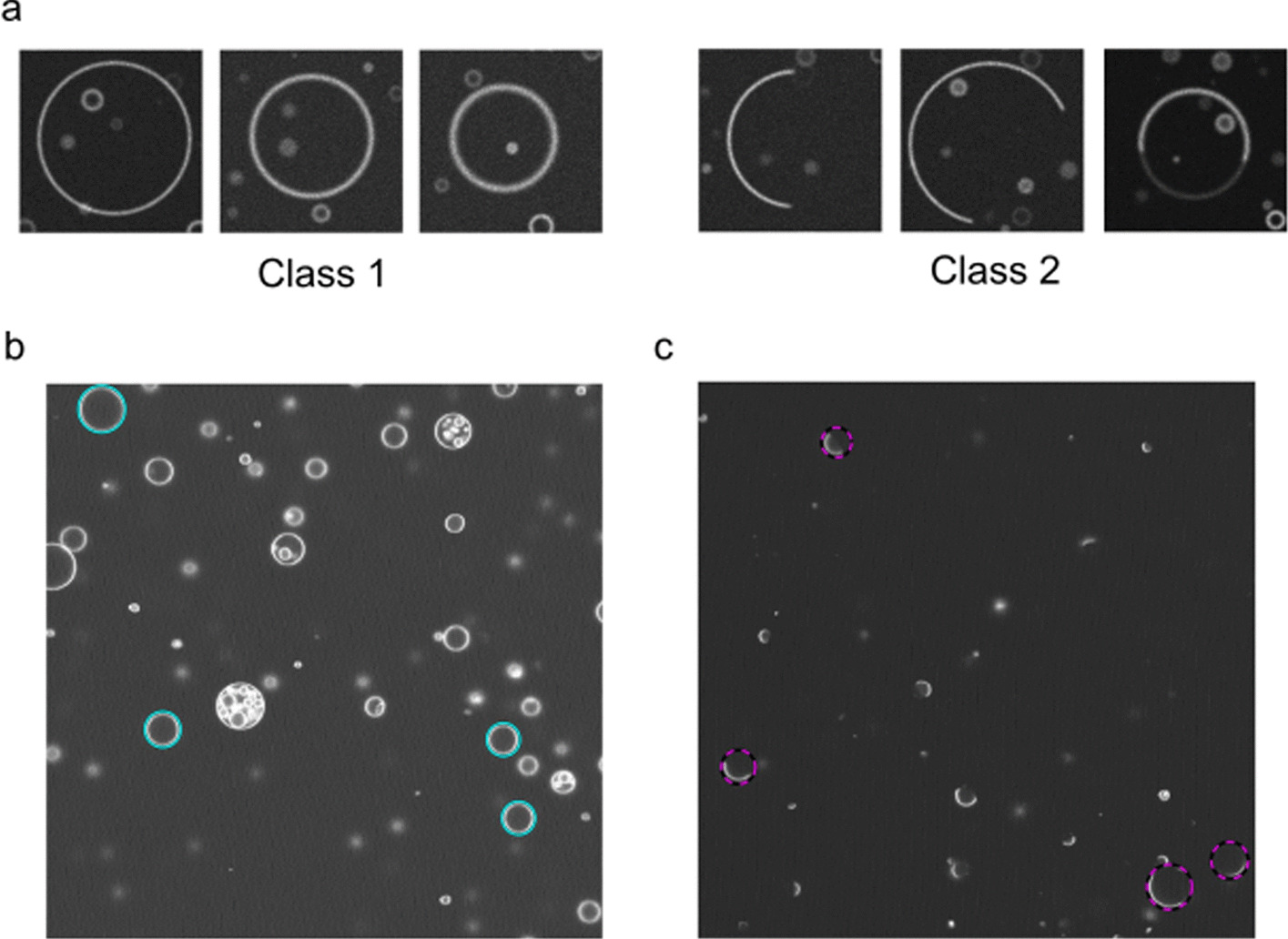
CNN-based phase state determination. **a** Typical vesicle images used to train the neural network. These are virtually simulated vesicle images. Class 1 represents vesicles that are uniform or homogeneous. Class 2 vesicle that are phase domain separated. **b**, **c** Example results of deep learning-based state analysis. Class 1—cyan, Class 2—magenta. The images are 127.3 μm in width and height

In the third version of the program, CNN was used for both vesicle selection and final phase domain state determination. Figure 10a shows typical example images of virtually created confocal fluorescence images of two different phase separation states, homogeneous and phase separated. TexasRed-DHPE fluorescence signal which is commonly used in vesicle labeling for domain separated vesicles was used for state determination, and the result shown is based on the TexasRed-DHPE fluorescence analysis. Vesicle compositions for homogeneous and phase separated vesicles were the same as the one used in program 2 state decision experiments except that 0.2 mol % TexasRed-DHPE was added. As shown in Fig. 10b, the program successfully selects desired vesicles and make final state analysis decision based on the trained CNN. Table 2 shows performance analysis by comparing CNN decision to human decision done the same ways as in Table 1. The same group of students researchers participated in Table 1 participated in survey in Table 2. The researcher who trained the network did not participate in this survey to avoid overfitting. As shown in the result, when 100 > n vesicles were analyzed from more than 30 images, mean accuracy of decision per vesicle reached 99.48% when compared with reference manual analysis. The performance can be considered as comparable to the human analysis for the purpose of final state decision.

**Table 2:** Performance of state determination by CNN classification

Type	Total number of GUVs analyzed (n)	Number of GUVs domain separated	Number of GUVs uniform	Mean accuracy of state decision (%)
Researcher 1	116	40	76	(Reference)
Researcher 2	116	37	78	(Reference)
Researcher 3	116	41	75	(Reference)
Researcher 4	116	40	76	(Reference)
Researcher 5	116	40	76	(Reference)
CNN Analysis	116	40	76	99.48

## Discussion

We conducted an intelligent fluorescence image analysis based on whole z-stack images of GUVs assisted by trained CNN. Our approach is unique in that it can effectively select the desired vesicles to analyze from a population of vesicles and perform fluorescence intensity-based calculation or CNN based decision on various pieces of information, such as the amount of lipid–protein interaction and the phase state of vesicles. Our method can detect vesicles from relatively low signal-to-noise ratio samples, and it can easily be expanded to various applications of multi-channel intensity analysis. We also showed that the method of automated detection can be used for protein droplet analysis, which we believe will be especially useful when studying the interaction between lipid vesicles and protein phase droplets. CNN-based classification could successfully recognize vesicle types for the purpose of selecting vesicles with a desired morphology in program 2. CNN classification also was able to determine and classify the phase domain separation state of the vesicles in program 3. To the best of our knowledge, this is the first implementation of deep learning to the problem.

Deep learning is potentially a very powerful approach because it can classify very complicated classes as long as an artificial neural network is well trained. However, its performance strongly depends on the quality and amount of training image sets, so a reasonable definition of classes with image samples spanning many variations of each class will be important for the training and validation accuracy. Due to the nature of raw image feeding for the CNN training, when the trained network did not successfully work as desired, its false classification was often difficult to rationalize which makes it difficult to plan what to specifically enhance in the training set other than increasing the number of images and augmenting them. For example, for human eye, determining phase separation state is a very easy task, much easier than determining vesicles with good signal to noise ratio. However, we found training CNN for phase state decision was much more challenging than to training it to distinguish clearly defined images from hazy images.

As typical image analysis in the field requires a process in which researchers identify each individual entity and make a decision on the class of each, the application of the deep learning approach can greatly improve the efficiency and reliability of the analysis. This report also include experimental work on the example of inhibitor based detachment of proteins from the vesicles, concentration dependent cargo concentration in protein liquid droplets and phase separation state of ternary mixture vesicles including Ni-DGS lipids.

To note a limitation of the circular segmentation, if the vesicle shape is only slightly elliptical, vesicles will be still detected. If the shape is completely non spherical due to severe deformation, circular segmentation algorithm will not detect it properly, thus the vesicles are best analyzed in a stable equilibrium condition. If the vesicle shape is spherical but with protruding tubes, the vesicles will be segmented but the tubular parts will not be counted in the analysis.

The programs analyze spherical GUVs by going through each z-section image that is later interpreted for individual vesicles. There are a few limitations worth mentioning related to the interpretation of the spherical geometry. Our programs perform intensity based analysis and CNN based analysis under the assumption that each image represents a contour intensity of a z-section. However, the very top and bottom images of each GUV tend to show 2D depiction of a lipid membrane as if they are images of a planar lipid membrane. Experienced researchers may even use such information to better make a phase state decision as 2D membrane images often shown clear domain boundaries of phase separated vesicles. In our programs, the initial selection process (Step3 of each program from Fig. 1) was designed to exclude those top images for consistency. Program 1 does this by excluding images with high interior intensity, and program 2/3 do this by excluding images classified as hazy images or blurred contour intensities. This approach was very successful with our experimental images, but confocal images may be collected with different resolution setting and contrast depending on the magnification, pixels per distance, illumination intensity, optical filters and fluorophores used. Therefore, for the CNN based selection, we can imagine that a neural network trained in one setting might be less successful interpreting images collected in another setting even though general features should be still distinguishable. For this reason, it is recommended that a convolutional neural network is trained with sample images collected in the matching setting for the finest classification performance. Inclusion of top images for the state decision analysis may have some but minor effect as the overall state decision is based on the ratio of phase separated z-sections from the entire z-section images. It may also cause a small error in intensity analysis as top images do not represent contour intensities. For fluorescence intensity analysis, our programs perform analysis by each z-section, thus average intensity values of sections would mean per image values not strictly per lipid area values. For program 1, we designed it to calculate per pixel value weighted by total number of pixels considered for membrane fluorescence, and it calculates a representative average value of the whole spherical shape. For program 2 and 3, fluorescence intensity trace for each z-section images can be obtained, thus users can selectively use the valid intensity information for their purposes. Z-sections taken at different heights of a spherical vesicle represent fluorescence intensity coming from lipids at different angles thus technically not at an identical condition. It may cause a difference in area of lipid illuminated at different angles, and excitation efficiency may be a concern if the illumination involves polarization. Our programs function appropriately with proper parameters set as shown in the results section, but if any of our assumptions made in whole z-stack analysis is inappropriate for the user’s purposes, users can consider modifying the codes to only use the central z-section images for their analysis.

## Conclusion

We developed MATLAB based software to automatically detect, select and analyze the GUVs from confocal fluorescence images. We showed versions of incremental applications of CNN to the problem on vesicle selection and phase state decision. We tested and reported performance of the codes by performing related experiments to collect sample images for each case. We showed case studies of training CNN by experimentally collected images and also by virtually simulated images. We hope our report serves as an example to develop further methods to apply CNN to similar problems of analyzing fluorescence images.

### Availability and requirements

- Project name: GUVanalysis
- Project home page: https://github.com/lipidprotein/GUVanalysis
- Group website: http://lipidprotein.com
- For the compiled version software: http://lipidprotein.com/Downloads/
- Operating system(s): Windows, Linux, Mac OS
- Programming language: GNU Octave, MATLAB
- Other requirements: MATLAB add-ons, including the Deep Learning and Image Processing Toolbox to run the raw codes of program 2 and 3.
- License: GNU
- Any restrictions to use by non-academics: none

## Supplementary Information

**Additional file 1**. The document include detailed explanation of user parameters in the programs.

## Data Availability

Sample image data for the demo runs can be found in the project home page (https://github.com/lipidprotein/GUVanalysis) and the group website (http://lipidprotein.com/Downloads/). More images relevant to the presented data can be found in the Montclair State University Digital Commons website (https://digitalcommons.montclair.edu/data/9/). Sample images include vesicle images that pass the selection filters and that do not pass the selection filters for demonstration purposes of the software functionality. Raw codes and sample images of the programs in this article are publicly available from the specified project home page. Compiled standalone applications are available from the specified group website.

## Abbreviations

CHT: Circular Hough transformation
CNN: Convolutional neural network
DOPC: 1,2-Dioleoyl-sn-glycero-3-phosphocholine
DPPC: 1,2-Dipalmitoyl-sn-glycero-3-phosphocholine
GFP: Green fluorescence protein
GUV: Giant unilamellar vesicle
ITO: Indium tin oxide
Ni-DGS: 1,2-Dioleoyl-sn-glycero-3-[(N-(5-amino-1-carboxypentyl)iminodiacetic acid)succinyl]
SIM: SUMO interacting motif
SUMO: Small Ubiquitin-like modifier
TF-Chol: Top Fluor-Cholesterol
TR-DHPE: Texas Red-1,2-Dihexadecanoyl-sn-Glycero-3-Phosphoethanolamine
