# Supplementary material for: Intelligent fluorescence image analysis of giant unilamellar vesicles using convolutional neural network
Source: BMC Bioinformatics. 2022 Jan 21;23:48. doi: 10.1186/s12859-022-04577-2 (PMC8783447; doi:10.1186/s12859-022-04577-2)
Supplement: Supplementary file 1 — Additional file 1. The document include detailed explanation of user parameters in the programs. [file 12859_2022_4577_MOESM1_ESM.pdf]

Supporting materials for:

Intelligent fluorescence image analysis of giant unilamellar vesicles using convolutional neural network

Il-Hyung Lee[1*], Sam Passaro[1], Selin Ozturk[1], Juan Ureña[1], Weitian Wang[2]

[1]Department of Chemistry and Biochemistry, Montclair State University, Montclair, NJ 07043, USA, [2]Department of Computer Science, Montclair State University, Montclair, NJ 07043, USA

The file include:

Detailed explanation of user parameters in the programs.

This supporting information explains all the key parameters used in the original program. We suggest users try with the default demo values before making any adjustment. S1-1 Segmentation related parameters are the ones that need the most frequent and active adjustment, and all other parameters should be changed based on the setting of image collection and brightness of the fluorescence reporter.

S1. Parameters common to Program 1, 2 and 3.

S1-1. Segmentation related parameters (A_intensity.m, B_Phase.m, C_NeuralPhase.m)

```
radius_min = 20; %smallest radius of GUV to detect
radius_max = 100; %largest radius of GUV to detect
```

radius_min and radius_max determines the range of circular radius the Circular Hugh Transform segmentation will be searching for. Any circular fluorescence between radius_min and radius_max will be detected, and circles beyond the range will be neglected. Unit is in pixels. These parameters can be used to selectively analyze circular images of a certain size, but setting a range way too off from the expected range can cause false segmentation. The program will run in any values set, but setting up too wide range will lower the quality of segmentation and increase the computational cost. When we tested, pixel range from 10 to 200 worked generally okay, and we suggest the pixel range of 100 pixels or less for efficient segmentation. Example detection at various cases are shown below.

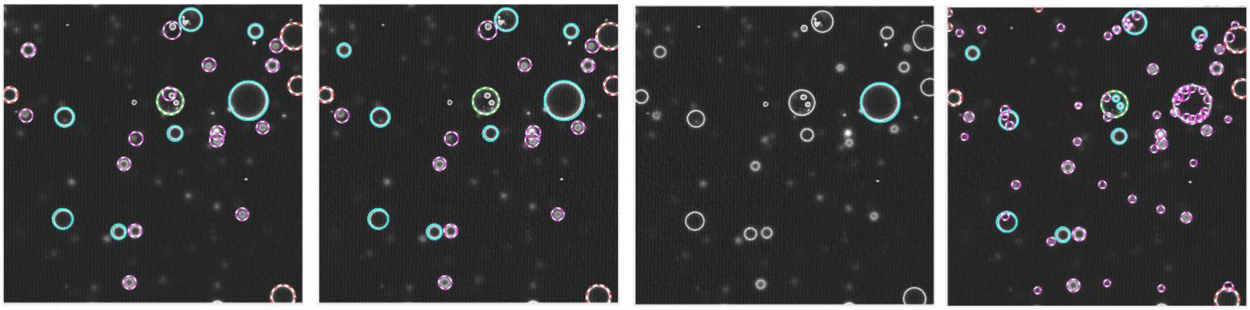

Radius 20 - 120 **Radius 20 - 200** **Radius 50 - 200** **Radius 10 - 50**

```
sensitivity = 0.90; %CHT fit sensitivity (0-1, 1 means free pass)
edge = 0.1; %CHT edge definition sensitivity (0-1, 1 means strong edge
needed)
```

These are two parameters used by Circular Hugh Transformation segmentation to decide what to count as circular object and what not to count. The segmentation is by voting process on likelihood of having a circular object of radius r at any pixel. Setting low sensitivity parameter will only pick up objects with high likelihood. (sensitivity = 1 means passing pretty much everything) Setting high edge parameter will only pick up objects with clearly defined edge. (edge = 1 means only the most strictly defined edge will be considered) Although each has its own definition, they are better understood as two related parameters to pick up more or less circular objects based on likelihood score. We suggest starting at sensitivity = 0.9, edge = 0.1 and incrementally change it by 0.05-0.1 until good detection is achieved.

We suggest starting with a single representative image stack and see if segmentation result looks reasonable. We recommend slightly over detecting instead of under detecting. It means the parameters should be set in a way that any obvious vesicular images appear in the detection result. It is okay to have

occasional false segmentation as such false detection will be naturally removed when reconstructing GUV stack. Always try the radius range first then determine sensitivity and edge together.

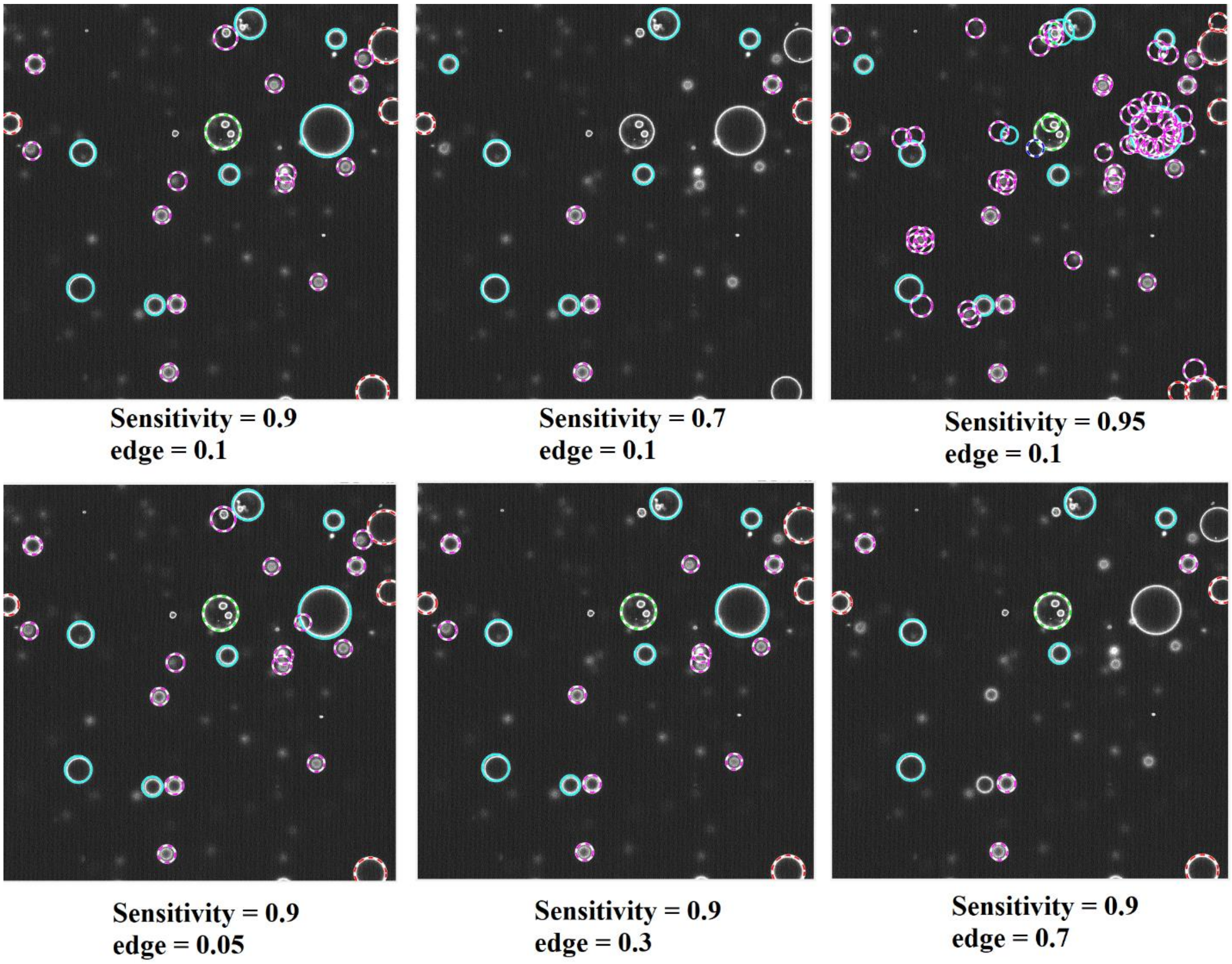

S1-2. Other parameters and options (A_intensity.m, B_Phase.m, C_NeuralPhase.m)

```
thres = 4; %number of pixels to count is within the fluorescence
thres2 = thres^2;
```

"thres" determines the distance threshold to use for intensity calculation. For example, if you set it thres = 4, it means any pixels that is within 4 pixels distance from the pixel right on the circle segmented will be counted as adjacent pixels and will be used for fluorescence intensity calculation. This value should be large enough to include smeared intensity by point spread function, but shouldn't be unnecessarily large which might count intensity coming from adjacent vesicles. Typically this value should be at least 2 pixels and can only be determined based on the nature of the optical setup and images acquired. We suggest users to study the Gaussian intensity thickness at different z-sections and use the greatest thickness encompassing the whole intensity profile as their parameters. thres2 is just thres x thres calculated to save number of computation.

```
trackDist = 5; %pixel distance to consider the same vesicles
```

The program will compare each z slice images and measure distance between two circles that are most likely to be from the same vesicle. If the distance is within “trackDist” it will be counted as the same vesicle, and will be combined into a GUV entity. This number should be large enough to account for small error in vesicle positions at different z section, but shouldn’t be too large which might combine two independent vesicle images as one. 5 is a reasonable number to start and it can be increased a bit as long as this number is reasonably smaller than distance between different vesicles.

This parameter can be used to accommodate the case where vesicle position moved slightly during the experiment of collecting z-stack images. If the movement is within this parameter value set, the program will tolerate the movement and will count those as a same vesicle.

```
initSkip = 1; %%number of images to skip (in case initial few are not good)
```

The program will skip initial “initSkip” images from the stack. You can set it zero if you want to analyze every image. You can use this if your first image usually does not contain useful information to analyze. (Possibly because you start your z-stack below the focus or your surface noise level is high etc.)

```
autoBackground = 1; %automatic estimation of background intensity by most
probable
                    %vlaue 1 to use mode estimation, value 2 for median
estimation
backgroundIntA = 0;  %manual input of the backgroudn intensity in case not
using auto estimation
backgroundIntB = 0;
```

Background noise is subtracted from the intensity calculation as explained as equation 1 in the main text. If you set the “autoBackground” as 1, it will use mode estimation to automatically calculate background intensity. (most probable value) Setting it 2 will use median value as background intensity. Users can set this zero and enter manually calculated value in “backgroundIntA” for the first channel and “backgroundIntB” for the second channel if there is any.

```
saveCircles = 1; %1 - on to save all circle figures as jpeg
% Important parameters to set for GUV stack analysis
```

Setting the logical parameter “saveCircles” on (1) will save figures for circle detection results as jpeg.

```
autoBinaryMask = 1; % 1 to use automatic binary mask thesholding 2 to use
manual mask value 0 to use raw images
maskThres = 0.5; % if not using automask, any intensity greater than this
avlue will be 1 and smaller will be 0
```

Setting the l “autoBinaryMask” as 1 will apply automatic binary masking for better segmentation. We suggest users just keep it as 1. If you prefer not to use it, you can set this 0. Users can set it 2 and enter manual intensity value to use for binary masking.

```
minNumStack = 5; % Minimum number of z-stacks needed to count as GUV (0 for
not using it)
```

Only GUV entities with at least “minNumStack” z-section images will be considered for final analysis. It means GUVs with 3 or 4 stack images will be excluded from the analysis when you set this parameter as 5. Set this value based on the nature of user’s data collection and size of vesicles.

```
isCloseStackfilt = 2; % 1 for filtering GUV Stacks that are too close
                      % 1 to remove both GUVs stuck 2 to reject only one with
less fitting fitness
```

You can use “isCloseStackfilt” to remove any GUV entities that are too close to each other. If you set this 1, you are excluding all GUVs that are possibly in too close proximity with another GUV. If you set this 2, it will look for two GUVs that are close to each other, and eliminate only one of the two. We suggest setting this value 0 to start, and set it on if needs arise.

S2. Parameters only relevant to Program 1

S2-1. Other parameters and options (A_intensity.m)

```
isMLVfilt = 1; % 1 for filtering MLVs 0 for no filer
MLVthres = 2.0; % 1.5 means outer membrane adjusted intensity should be at
least 150% inner membrane adjsuted intensity to pass
```

Logical parameter “isMLVfilt” can be set on (1) to compute intensity inside the vesicle and compare it to the average intensity around the circular fluorescence from the vesicular membranes. When users use this, only when average intensity on the membrane is “MLVthres” times greater than the average intensity inside the vesicle, the detected circular image will be considered for further computation. (Vesicles with too much nonspecific fluorescence due to multi-lamellarity will be excluded.) Users can set this off (0) if such selection is unnecessary.

S3. Parameters only relevant to Program 2 and Program 3

S3-1. Other parameters and options (B_Phase.m, C_NeuralPhase.m)

```
saveEachIntensity = 1; %1 - on to save all circle intensity history
(including GUV filtered ones)
```

Setting logical parameter “saveEachIntensity” on (1) will save intensity trace information as text files.

```
decisionP = 0.4; %Vesicles with decisionPx100% of phase separation will be
called phase separated.
```

Final phase state decision is made for each vesicle entity. When a vesicle contain 100% x “decisionP” section images, it will be called as a phase separated GUV. For example, if your vesicle has 10 z-section images, and 5 images show phase separated state, it will be classified as a phase separated vesicle because more than 0.4 x 10 images showed phase separation. 0.4 or 0.5 worked fine for general ternary vesicles, but if your vesicle domain area is relatively small, you might want to consider smaller value.

```
ifFilterEdgeVes = 1; %1 means vesicles partially detected at the edge will be
excluded for analysis.
                     % This should be on unless necessary.
```
Setting “ifFilterEdgeVes” on (1) will eliminate all vesicle images at the edges (incomplete circles).

```
isNRfilt = 1; % 1 for using neural decision to filter invalid GUV images
```
Setting “ifFilterEdgeVes” on(1)will perform CNN based image selection. You can set this off (0) if you just want to skip the selection process.

```
nrMargin = 1.2; % to multiply to make sure image contains the circle.
```
This parameter appears at the middle of the script, and we suggest users do not change it unless necessary. It determines the area of image relative to the size of circular segmentation to use as an input image in CNN decision analysis. For example, if your circle is 100 pixels in diameters, 120 x 120 pixels image will be used as a neural input.

Loading neural network is done by loading saved matlab file which include variables saved after CNN training. Users can save their own neural network and load them. Just be aware in the default script, name “net” is used to refer to CNN network for vesicle image selection, and “net2” is used to refer to CNN network for phase state decision.

S4. Parameters only relevant to Program 2

S4-1. Other parameters and options (B_Phase.m)

```
phasDecision = 0.1; %variable to make decision about discontinuity around
fluorescence contour
                    %It mens Lowintensity <= Highintensity x 200% x
phasEcision
                    %determine this based on manual examination of per pixel
intentisy of two
                    %different phases
```
In intensity trace based phase separation decision, the program will calculate average intensity around the circular periphery of the fluorescence to create intensity trace. When intensity increases or drop by “phasDecision” from the previous trace, the program will count it as discontinuity or signature of phase domain boundary. You can determine this value based on general partition coefficient of your reporter molecule between two domains. If your reporter has clear contrast, set this value large enough to avoid false positive decision on phase separation. We suggest starting with 0.1 to test on control images including vesicles with phase separation and no separation, and adjust it to the value that does not make false decision.

The program distinguishes existence of single domain (two boundaries) and more than single domain (more than two boundaries) which is classified as “Multi” domains. If existence of such multiple domain existence is relevant to user’s experiment, it can be used, but otherwise, both “Phase” and “Multi” mean phase separation.

S5. Parameters only relevant to Program 3 (C_NeuralPhase.m)

S4-1. Parameters for virtual image creation (run_GUV.m)

```
Npts=[504 504 504]; %dimension of the ground truth
confpix=[2 2 8]; %coversion ratio between ground trugh and virtual image
confpsf=[4 4 7]; %dimension of point spread function in pixels
```

"Npts" determines x, y, z dimension of the original ground truth to be generated. Although technically there is no limit to this number, we suggest try 504x504x504 and do not go too much beyond that. This is because that the three dimensional space can take up memory very easily and bigger project will be burdensome in any single computing device. "confpix" determines the ratio between ground truth and final confocal image created. For example value 2 in x means original 504 ground truth will be 504/2 = 252 pixels in x. "confpsf" is dimension of point spread function of x,y,z as an input to virtual confocal image generation. Greater number means bigger point spread function and more spread of fluorescence intensity in confocal image. Fractional numbers can be used for "confpsf" if desired. We suggest users try creating a few virtual images to see if the resulting images look reasonable if they wish to vary the scale.

```
R = 170 + rand() * 60; %radius of the ground truth vesicle
L = 1.0 + rand(); %bilayer thickness of the ground truth
```

"R" is for radius of the vesicle created. For training purpose, it is the best have various radius. "L" is for bilayer thickness of the original ground truth image. Larger L value means thicker fluorescence intensity in the final image.

```
value = 1000 + 1000*rand();
value2 = round(value* (0.5 * rand()));
```

"value" and "value2" determines intensity of two different domains. If the vesicle is not phase separated, only the first "value" will be used. It is the relative intensity ratio between "value" and "value2" that is an important parameter to consider for training purposes.

S4-2. Parameters for ground truth generation (createGUVimages.m and createGUVimages_binary.m)

```
% Center position shifter x-y on/off (to prevent all images exactly
% centered, choosing this will move the center of the vesicle in x-y.
isCenterShift = 1;
% amount of shift, will be determined randomly from +/- zero to this value.
% Avoid Radius + shift being greater than 1/2 of the dimension
centerShift = 20;
```

If you want each vesicle generated to have slight shift in its central coordinate, you can turn "isCenterShift" on (1) and set the "centerShift" number as maximum random variation in pixels. This can be done in Matlab function of augmented training too, so if not desired, users can turn this off.

```
% true speckel noise addition on/off
isNoise = 1;
% how much to add 0.01 means 1% of the pixesl will be added as spekcle noise
noiseRatio = 0.00001;
% Noise intensity, noise intensity will be randomized from zero to this
% value
noiseIntensity = value;
```

"isNoise" can be turned on (1) to add speckle noise to the original ground truth image. "noieRatio" determines how many speckles to add. "noiseIntensity" will be used as groundtruth intensity of the noise. Our training experience suggest having speckle noise is not critical in the training outcome, so it can be turned off (0) if not needed.

```
% small vesicle addition on/off
isVesicle = 1;
% how many vesicles to add per image stack
vesicleNumber = 100;
% Vesicle intensity,intensity will be randomized from zero to this value
vesicleIntensity = value;
% Vesicle radius. will be randomized from zero to this value
vesicleRadius = 30;
```

Having "isVesicle" on (1) will add tiny vesicles into the images as simulated noise. Real images may have similar vesicles inside or outside the vesicle of interest. "vesicleNumber" determines number of small vesicles to add and "vesicleIntensity" is max fluorescence intensity value of small vesicles and "vesicleRaidus" is max radius of small vesicles in pixels. We suggest have this feature on to add variations to training images.

S4-3. Parameters for confocal image generation (cgn_run_images.m)

```
save=1;
```

Having "save" on(1) will save created images. It should be on unless running for other purposes.

```
display=0;
```

Having "display" on (1) will show images generated as they are generated. Users can use this to get the sense of image creation, but for large quantity work, having display on will significantly slow down the work.

```
plot=0;
```

Having "plot" on (1) will show overall segmentation and plotting result in an organized plot. Similar to "display" it should be off for a large computational work.

S4-5. Parameters for image conversion for neural network training (PixelConvertDenoise.m)

```
pixel = 50
```

You can change "pixel" if you want to change x, y dimension of the converted images. Since it changes the input for the CNN training itself, consider changing it only if you are planning to try different image size as an input which can greatly affect the training efficiency.

```
maskThres = 65535 * triangle_th(lehisto, 11107);
```

"maskThres" is the value that will be subtracted as a baseline value during the conversion. If you want to set this differently, users can enter any value or equation here. Default is to use triangular thresholding.

S4-6. Parameters for CNN training (Train.m)

```
inputSize = [50, 50, 1];
```

"inputSize" determines the dimension of input image. Only change if users want to try different dimension which can greatly affect training efficiency and output.

```
pixelRange = [-10 10];
```

"pixelRange" is used to randomly shift center of image in the range for augmented CNN training (more variation for effect CNN training).

```
scaleRange = [0.9 1.1];
```

"scaleRange" is used to randomly rescale the images within the range for augmented CNN training (more variation for effect CNN training)

The rest of the sentences for CNN structure follows the general MATLAB definition and it is explained in the main text. Final network is saved by saving the current variables using the save command.
